# Comparison of PESA and MESA techniques for men with obstructive azoospermia

**DOI:** 10.1016/j.urolvj.2019.100010

**Published:** 2019-08-26

**Authors:** Joshua Bitran, Premal Patel, Ranjith Ramasamy

**Affiliations:** Department of Urology, University of Miami Miller School of Medicine, 1120 NW 14th Street, Room 1560, 33136, United States

**Keywords:** PESA, Percutaneous Epididymal Sperm Aspiration, MESA, Microsurgical Epididymal Sperm Aspiration, Obstructive Azoospermia

## Abstract

**Design:**

Video presentation.

**Setting:**

University hospital.

**Patient(s):**

A 53-year-old male presents with a history of a vasectomy performed 7 years prior. His wife is 36 years-old and requests sperm extraction for in-vitro fertilization. On examination, his testicles were 20 cc bilaterally with a serum follicle-stimulating hormone of 5.3.

**Intervention(s):**

Percutaneous Epididymal Sperm Aspiration (PESA) and Microscopic Epididymal Sperm Aspiration (MESA)

**Main outcome measure(s):**

Intraoperative technique highlighting the main steps for performing PESA and MESA, complications, and sperm retrieval outcomes.

**Result(s):**

This video highlights the technique for performing both PESA and MESA. We demonstrate complications and outcomes associated with both procedures. Both PESA and MESA are viable options for sperm retrieval with varying complications and sperm quality outcomes.

**Conclusion(s):**

We demonstrate how to perform both PESA and MESA. Both are effective means for obtaining sperm for in-vitro fertilization with differences in technique, equipment required, complications and sperm quality outcomes.

## Supplementary Material

Video

